# Risk of Myocardial Infarction in Parents of HIV-infected Individuals: a population-based Cohort Study

**DOI:** 10.1186/1471-2334-10-169

**Published:** 2010-06-14

**Authors:** Line D Rasmussen, Lars H Omland, Court Pedersen, Jan Gerstoft, Gitte Kronborg, Janne Jensen, Niels Obel

**Affiliations:** 1 Department of Infectious Diseases, Odense University Hospital, Odense, Denmark; 2 Department of Infectious Diseases, Copenhagen University Hospital, Rigshospitalet, Denmark; 3 Department of Infectious Diseases, Copenhagen University Hospital, Hvidovre, Denmark; 4 Department of Infectious Diseases, Kolding Sygehus, Kolding, Denmark

## Abstract

**Background:**

Previous studies have indicated an increased risk of myocardial infarction (MI) in HIV infected individuals especially after start of highly active antiretroviral therapy (HAART). It is however controversial whether the increased risk of atherosclerotic disease is exclusively associated with the HIV disease and HAART or whether life-style related or genetic factors also increase the risk in this population. To establish whether the increased risk of myocardial infarction in HIV patients partly reflects an increased risk of MI in their families, we estimated the relative risk of MI in parents of HIV-infected individuals.

**Methods:**

From the Danish HIV Cohort Study and the Danish Civil Registration System we identified the parents of all HIV-infected patients born in Denmark after 1952 in whom a Danish born mother was identifiable. For each HIV patient, 4 matched population controls and their parents were identified. Cumulative incidence functions were constructed to illustrate time to first MI of the parents as registered in the Danish National Hospital Registry. Incidence rate ratios (IRR) were estimated by Cox's regression analyses. Due to the confidential type of the analysed data the study was approved by the Danish Data Protection Agency.

**Results:**

2,269 mothers and 2,022 fathers of HIV patients as well as 9,076 mothers and 8,460 fathers of control subjects were identified. We observed an increased risk of MI in mothers of HIV patients (adjusted IRR, 1.31; 95% CI: 1.08-1.60). The strongest association was seen in case the offspring was infected heterosexually (adjusted IRR, 1.59; 95% CI: 1.07-2.35) or by IV drug abuse (IVD) (adjusted IRR, 1.63; 95% CI: 1.02-2.60). In fathers of HIV patients the risk of MI was only increased if the offspring was infected by IVD (adjusted IRR, 1.42; 95% CI: 1.01-2.00).

**Conclusion:**

Mothers of HIV-infected patients have an increased risk of MI. We presume that this stems from family related life style risk factors, some of which may also influence the risk of MI in HIV-infected patients.

## Background

The introduction of highly active antiretroviral therapy (HAART) has reduced mortality and morbidity dramatically in HIV-infected patients [1]. HAART has, however, been shown to cause a number of metabolic changes and potentially atherosclerotic complications [2,3]. Some studies have indicated an increased risk of myocardial infarction (MI) even in HIV patients not on HAART [4,5]. In a study from the Danish HIV Cohort we found a relative risk of MI of 1.39 in patients not on HAART when compared to the background population [4]. The risk was further increased following HAART initiation (adjusted relative risk 2.12), and the risk did not increase further during the first 8 years of HAART. This was in contrast to the DAD-study, which concluded that the risk of MI increased progressively with the duration of HAART exposure [6]. Initially the main focus was on use of protease inhibitors, but in 2008 the DAD study found that current exposure to abacavir and didanosine was associated with an increased risk of coronary events [7]. It is however still controversial whether the increased risk of atherosclerotic disease is exclusively associated with the HIV disease and HAART or whether it is also increased due to life-style related or genetic factors.

A family history of atherosclerotic disease is associated with an increased risk of MI [8]. The association most likely is mediated not only through genetic factors, but also through shared socio-economic and environmental factors affecting life-style e.g. smoking, diet, body mass index, physical activity as well as the use of medical services. Previous studies have adjusted the estimated risk of MI for known cardiovascular risk factors [4-6,9-13], estimated and compared the distribution of risk factors in HIV patients with that of the background population [6,14-16] or simply excluded patients with risk factors from their study [17]. These studies though may be hampered from residual confounding or confounding due to non-measured factors. As a measure of these unaccounted for confounders we aimed to estimate the risk of MI in parents of HIV-infected individuals. We therefore conducted a population based, nationwide cohort study of the risk of MI in the parents of HIV-infected patients compared to the risk in parents of population controls.

## Methods

**Figure 1:**
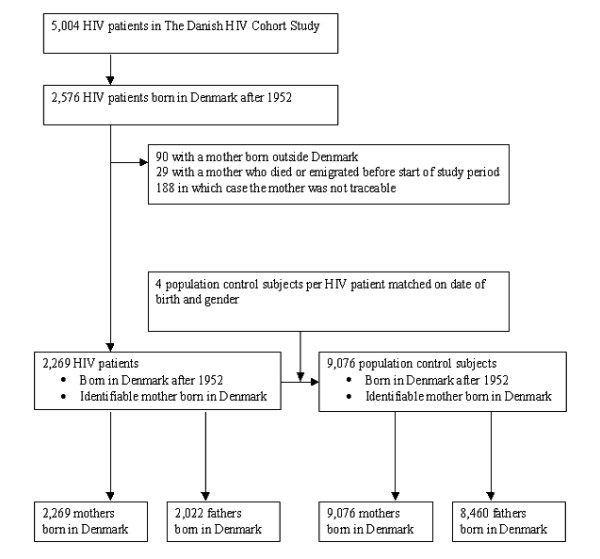
**Summary of the study design**.

The study design is presented in figure 1.

### Setting

As of 1 January 2007 Denmark had a population of 5.4 million (Statistics Denmark, http://www.statbank.dk), with an estimated HIV prevalence of 0.07% among adults [18]. Medical care, including antiretroviral treatment, is tax-paid and provided free-of-charge to all HIV-infected residents of Denmark.

### Data sources

We used the unique 10-digit civil registration number assigned to all individuals in Denmark to link the data sources described below.

#### The Danish HIV Cohort Study

The Danish HIV Cohort Study, described in detail elsewhere, is a nationwide, prospective, population-based cohort study including all Danish HIV-infected patients treated at Danish hospitals since 1 January 1995 [18]. The data are updated on a yearly basis and include amongst others demographics, route of infection, all CD4 counts, viral loads and antiretroviral treatment.

#### The Danish Civil Registration System

The Danish Civil Registration System was established in 1968 and stores information of vital status, residency as well as immigration and emigration on all Danish residents [19]. A 10-digit personal number (Central Person Registry [CPR] number), assigned at birth, uniquely identifies each citizen. Since 1 January 1969 the registry also included identification of parents still alive at this date.

#### The Danish National Hospital Registry

The Danish National Hospital Registry was established in January 1977 and records all discharge diagnoses according to ICD-8 and ICD-10 codes, all operation codes according to IOC codes and since 1995 all hospital outpatient visits for patients treated in Danish hospitals (The International Classification of Diseases 8^th ^revision until the end of 1993 and here after 10^th ^revision - ICD-9 has never been used in Denmark) [20].

### Definition of MI

The date of the myocardial infarction was defined as the first date an individual was registered in the Danish National Hospital Registry with one of the following ICD-8 or ICD-10 diagnosis: 410.9, 410.99, I21.0-21.9.

### HIV-infected patients and general population control cohort

The HIV patients were identified from the Danish HIV Cohort Study (figure 1). The index date in these patients was defined as 1 January 1995 or the date of the HIV diagnosis which ever came last. We identified all HIV-infected patients who: 1) were registered in the Danish Civil Registration System, 2) born in Denmark after year 1952 (the registration of parents in the Danish Civil Registration System is incomplete for individuals born before 1953), 3) had a mother born in Denmark, who were identifiable in the Danish Civil Registration System and 4) living in Denmark on the index date.

For each of the HIV-infected patients we identified 4 age- and gender matched population control subjects from the Danish Civil Registration System who: 1) were registered in the Danish Civil Registration System, 2) born in Denmark after year 1952, 3) had a mother born in Denmark, who were identifiable in the Danish Civil Registration System and 4) living in Denmark on the index date of the matching HIV patient.

### Study populations (parents)

We identified all Danish born parents of the HIV-infected patients and population control subjects who were registered in the Danish Civil Registration System (figure 1). Index date of the parents was defined as the start of the Danish National Hospital Registry (January 1 1977) or date of birth of the offspring which ever came last.

### Statistics

We assessed the risk of MI in parents of HIV-infected patients as well as parents of the control cohort subjects. Time was calculated from index date to the first of the following: - date of MI, emigration, death, lost to follow up or 1 June 2008. We used cumulative incidence function to illustrate time to first MI registered in the Danish National Hospital Registry, recognizing death as a competing risk [21]. Incidence rate ratios (IRR) and 95% confidence intervals for MI as estimates of relative risks were calculated using Cox proportional-hazards regression. Results were stratified on mothers and fathers. Further the IRR was adjusted for: age at start of observation (continuous variable) as well as year of birth of the parent divided into the following decades: - before1920, 1920-1930, 1930-1940, 1940-1950, after 1950.

Cox regression analyses were stratified on route of HIV transmission in the HIV patient (homosexual, heterosexual or intravenous drug abuse (IVD)).

Statistical analyses were performed using SPSS version 17.0 and R version 2.8.1. The study was approved by the Danish Data Protection Agency (Denmark has no Institutional review boards).

## Results

Of the 5,004 HIV patients in the Danish HIV Cohort Study 2,576 were born in Denmark after 1952. Of these 2,576 patients, 90 had a mother born outside Denmark, 29 mothers had died or emigrated from Denmark before the start of the study period (1 January 1977) and 188 had mothers, who were untraceable in The Danish Civil Registration System leaving 2,269 individuals in the study population (figure 1). For these individuals we identified 9,076 population controls. The demographics of the two populations are shown in table 1.

**Table 1:** Characteristics of HIV-infected patients and population controls.

	HIV-infected patients	Control subjects
Number of study subjects	2,269	9,076
Males (number (%))	1,895 (83.5)	7,580 (83.5)
Median age at index date (years (IQR))	33.8 (29.2-38.7)	33.8 (29.2-38.7)
Route of infection:		
Homosexual (number (%))	1,205 (53.1)	
Heterosexual (number (%))	555 (24.5)	
Injection drug abuse (number (%))	336 (14.8)	
Other/unknown (number (%))	173 (7.6)	

We identified 2,269 mothers and 2,022 fathers of HIV-infected patients as well as 9,076 mothers and 8,460 fathers of control subjects (figure 1 and table 2). The parents of the HIV-infected patients were almost the same age as that of the population controls (Table 2). Due to the study design Denmark was registered as country of birth in all parents. The median time of follow up was almost 30 years in all groups and very few subjects were lost to follow up.

**Table 2:** Characteristics of parents of HIV-infected patients and population controls.

	Mothers of		Fathers of	
	HIV-infected patients	Control subjects	HIV-infected patients	Control subjects
No of parents	2,269	9,076	2,022	8,460
Median age at index date (years (IQR))	38.3 (32.3-44.9)	38.7 (32.4-45.3)	41.5 (34.6-49.1)	41.9 (34.6-49.2)
Number in whom observation time started after 1. January 1977 (%)	197 (7.9)	699 (7.7)	200 (8.8)	773 (8.1)
First admission with MI after index date:	129 (5.7)	434 (4.8)	257 (12.7)	1,085 (12.8)
Emigration during follow-up, N (%)	19(0.8)	35 (0.4)	18 (0.9)	44 (0.5)
Lost to follow-up, N (%)	0 (0.0)	1 (0.0)	1 (0.0)	3 (0.0)
Duration of follow up				
Duration of follow-up, person years	61,900	256,033	49,443	213,883
Duration of follow-up, median years (IQR)	31.4 (25.9-31.4)	31.4 (28.3-31.4)	29.9 (18.7-31.4)	31.0 (20.6-31.4)
Overall risk of MI, per 1,000 PYR (95%CI)	2.08 (1.75-2.48)	1.70 (1.54-1.86)	5.20 (4.60-5.87)	5.07(4.78-5.38)

During the study period the overall risk of MI was 2.08/1,000 PYR (95% CI: 1.75-2.48) versus 1.70/1,000 PYR (95% CI: 1.54-1.86) for mothers of HIV patients and control subjects and 5.20/1,000 PYR (95% CI: 4.60-5.87) versus 5.07/1,000 PYR (95% CI: 4.78-5.38) for fathers of HIV patients and control subjects (Table 2).

**Figure 2:**
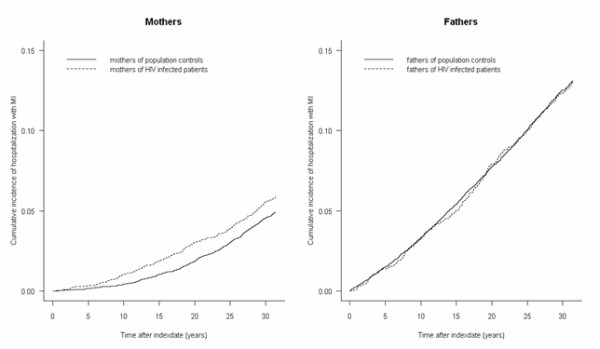
**Cumulative incidence of hospitalization with MI in mothers and fathers**.

Risk of MI was significantly higher in mothers of HIV-infected patients compared to the control subjects (adjusted relative risk, 1.31; 95% CI: 1.08-1.60) (Table 3 and Figure 2). The risk of MI was highest in mothers of HIV patients who reported heterosexual contact (adjusted relative risk, 1.59; 95% CI: 1.07-2.35) and IVD (adjusted relative risk, 1.63; 95% CI: 1.02-2.60) as route of HIV infection (Table 3).

**Table 3:** Risk of MI in parents of HIV- infected patients and population controls.

	**Mothers**:		**Fathers**:	
	Unadjusted IRR (95% CI)	Adjusted IRR (95% CI)	Unadjusted IRR (95% CI)	Adjusted IRR (95% CI)
All parents	1.25 (1.03-1.52)	1.31 (1.08-1.60)	1.03 (0.90-1.18)	1.04 (0.901.19)
Parents of homosexual patients	1.11 (0.84-1.48)	1.10 (0.82-1.47)	0.98 (0.81-1.18)	0.94 (0.78-1.13)
Parents of heterosexual patients	1.46 (0.99-2.15)	1.59 (1.07-2.35)	1.02 (0.77-1.35)	1.07 (0.81-1.41)
Parents of IV-drug addicts	1.31 (0.82-2.08)	1.63 (1.02-2.60)	1.31 (0.94-1.83)	1.42 (1.01-2.00)

Risk of MI in fathers of HIV-infected patients was almost similar to that of control subjects (adjusted relative risk, 1.04, 95% CI: 0.90-1.19) (Table 3 and Figure 2). However having an offspring reporting IVD as route of infection, seemed to substantially increase the risk of MI in the fathers (adjusted relative risk, 1.42; 95% CI: 1.01-2.00).

## Discussion and Conclusion

In this population based nation wide cohort study we found an increased risk of MI in mothers of HIV-infected patients compared with mothers of the general population. The increased risk of MI was mainly seen in mothers of HIV patients reporting to be infected heterosexually or by IVD. In the fathers the increased risk of MI was only seen in case the offspring reported IVD as route of HIV infection.

The strength of our study is the nationwide population based design as well as a long and complete follow-up and the access to valid data on family members. We included parents of HIV patients and parents of a well-matched population control cohort. Further we were able to stratify our results on several important factors as parental gender, route of HIV transmission as well as calendar periods. As immigrants may retain higher risk of a disease due to susceptibility periods during childhood for some diseases as well as the potential effect of acculturation, immigrants were excluded from our study [22,23]. In the start of the epidemic in the 1950s coronary heart disease was associated with high socio-economic status, but over time became a disease of the lower social classes. This, as well as the fact that treatment and prevention of ischaemic heart disease has changed considerably over time, makes control of calendar periods essential [8,24]. We are not aware of other studies with a similar design.

Our study has some limitations. To identify patients with the MI diagnosis we relied on hospital registry based discharge diagnoses, which may not be entirely accurate and includes a risk of incorrectly registered diagnoses due to pre-hospital death. Importantly though we used the same source of data to ascertain MI for all study objects. Furthermore identification of MI on behalf of discharge diagnoses has previously been shown to be valid [25]. We could not adjust for specific indicators of social class and socio-economic status such as parental education, occupation, family income, housing conditions, maternal marital status or illegitimacy. However, reported route of HIV infection in the offspring may act as a surrogate marker for socio-economic status.

Denmark harbours well-designed registries, which provide full access to data on all inhabitants concerning vital status and hospital contacts. As the Danish National Hospital Registry was not initiated until January 1977, some of the study subjects (i.e. the parents) might have had MI prior to study inclusion. These subjects are at increased risk of MI and ideally should have been excluded. However, as the Danish National Hospital Registry does not allow us to distinguish between first and subsequent episodes of MI in case the first MI occurred before 1977, we have no means of excluding these patients. Nevertheless, this phenomenon could hardly have biased our results substantially as the study subjects were young at inclusion, which makes previous episodes of MI unlikely. Further, age at study inclusion did not differ markedly between the compared groups (mothers versus mothers, fathers versus fathers) and therefore the proportion of post MIs misclassified as primary MI probably did not differ between groups. The relative risk measures therefore would remain rather unchanged even in case of inclusion of some post MI subjects. Parents were not included in the study if they had never been registered as parents in the Danish Civil Registration System, or had died prior to year 1977 as diagnoses of MI in the Danish National Hospital Registry were first available from that year. In 188 cases (7.6%) we were not able to trace the mothers in The Danish Civil registration System. We presume that the bias this may introduce is equivalent in the patient/control populations and does not influence our estimates.

In general the absolute risk of MI is higher in males than in females. However, in our study the increase in relative risk of MI was substantially higher in the mothers compared to the fathers. Several factors may explain this observation. First low socio-economic class, associated with particular lifestyle patterns, has been described as a stronger risk factor of MI for women than for men [26]. Second the offspring may be more prone to share life style characteristics with their mothers than their fathers possible due to a preponderance of single family homes with mothers raising the children. And third the absolute risk of MI was almost three times higher in the fathers compared to the mothers. Therefore a minor increase in absolute risk of MI in the mothers will result in a greater increase in relative risk in this population compared to the fathers.

Although ischaemic heart disease manifests itself in adulthood, the disease and underlying atherosclerosis, develops throughout life [23]. An increased risk of MI has previously been shown to be associated with lower social class in adulthood [22,23,27]. However, adverse circumstances during pregnancy and childhood, including poor socio-economic conditions, seem to be important determinants of risk of adult cardiovascular disease. These risk factors are not merely due to the continuity of disadvantage throughout life [8,23,24,28]. In a study by Kaplan et al it was suggested that HIV-infected individuals from economically disadvantaged populations bear a disproportionate burden of risk factors that might explain the association of MI and HIV [27]. In addition to this it has previously been established that the prevalence of smokers are consistently higher in HIV patients than in age-matched controls [14,27,29-31]. We cannot exclude that smoking may be a major contribution to the increased risk of MI in parents of HIV patients acting independently of other risk factors.

Our results showed a strong association between IVD as route of HIV infection in the offspring and risk of MI in both mothers and fathers of HIV patients. A strong association between initiation of IVD in the offspring and adverse socio-environmental factors in childhood has been described and this association probably explains the association observed in our study [32,33].

Our study cannot unambiguously discriminate to what extend genetic and environmental factors exert their impact on risk of MI in the parents of HIV patients. But the findings imply that shared socio-economic disadvantages to some extent explain the excess risk of MI observed in the mothers. The genetic element in MI is not to be questioned but to our knowledge no genetic factors have been described, which is associated with increased risk of both HIV acquisition and MI. However, health-risk behaviours are frequently interrelated [34] and tendency to "risk taking behaviour" has been explained as a result of a complex combination of social (e.g. socio-economic and environmental factors), genetic and developmental factors [35-39]. In that case initiating IVD, sharing needles and having sex without barrier protection as well as heavy smoking, alcohol consumption, inappropriate diet and physical inactivity, could all be a result of the same genetic predisposition.

The increased risk of MI in HIV infected individuals has mainly been ascribed to the HIV infection per se and exposure to HAART [4-7,11,12,15-17,40]. Additionally it has been questioned whether a HIV-positive status simply serves as a marker for differences in the prevalence of conventional risk factors such as smoking [29]. We found an increased risk of MI in mothers of HIV patients of almost the same order (IRR: 1.31) as that seen in their HIV infected offspring when not on HAART (IRR: 1.39) [4]. Our data therefore indicate that a main proportion of the increased risk of MI in these mothers and their HIV-infected offspring may stem from the same family related life style factors.

The implications of our findings extend beyond establishing the risk of MI in the parents of the HIV infected patients. Recent studies suggest premature aging, accelerated cognitive decline, increased risk of non-AIDS associated cancers and osteoporosis in HIV infected patients despite successful HAART [41]. These studies may potentially be hampered by substantial influence from family related risk factors as well. Thus our data indicate that the association between risk for common diseases and HIV exposure may make it difficult to definitively establish HIV or antiretroviral drugs as causal risk factors for comorbidity in HIV infected patients.

In conclusion, mothers of HIV-infected patients have increased risk of MI. We presume, that the increased risk is related to family related life style risk factors, some of which may also influence the risk of MI in the HIV-infected patients. Thus, a major fraction of the increased risk of MI observed in HIV-infected patients may stem from non-HIV-related factors.

## Competing interests

NO has received research funding from Roche, Bristol-Myers Squibb, Merck Sharp & Dohme, GlaxoSmithKline, Abbott, Boehringer Ingelheim, Janssen-Cilag and Swedish Orphan. CP has received research funding from Abbott, Roche, Bristol-Myers Squibb, Merck Sharp & Dohme, GlaxoSmithKline, Swedish Orphan and Boehringer Ingelheim. JG has received research funding from Abbott, Roche, Bristol-Myers Squibb, Mecrk Sharp & Dohme, Pharmasia, GlaxoSmithKline, Swedish Orphan and Boehringer Ingelheim. LDR, LHO, CP, GK and JJ report no conflicts of interest.

## Authors' contributions

Conception and design: LDR, LHO, NO. Analysis and interpretation of the data: LDR, LHO, NO. Drafting of the article: LDR Critical revision of the article for important intellectual content: LDR, LHO, CP, JG, GK, JJ, NO. Provision of study materials or patients: CP, GK, JG, JJ, NO. Statistical expertise: LDR, LHO, NO. Obtaining of funding: NO. Administrative, technical, or logistic support: LDR, NO. Collection and assembly of data: CP, GK, JG, JJ, NO. All authors read and approved the final manuscript.

## Pre-publication history

The pre-publication history for this paper can be accessed here:

http://www.biomedcentral.com/1471-2334/10/169/prepub
